# Preterm Birth during Influenza Season Is Associated with Adverse Outcome in Very Low Birth Weight Infants

**DOI:** 10.3389/fped.2016.00130

**Published:** 2016-11-30

**Authors:** Christoph Härtel, Alexander Humberg, Dorothee Viemann, Anja Stein, Thorsten Orlikowsky, Jan Rupp, Matthias V. Kopp, Egbert Herting, Wolfgang Göpel

**Affiliations:** ^1^Department of Pediatrics, University of Lübeck, Lübeck, Germany; ^2^Department of Neonatology, Hanover Medical School, Hanover, Germany; ^3^Department of Pediatrics I, University of Duisburg-Essen, Duisburg, Germany; ^4^Department of Neonatology, University of Aachen, Aachen, Germany; ^5^Department of Infectious Diseases and Microbiology, University of Lübeck, Lübeck, Germany; ^6^Airway Research Center North (ARCN), German Lung Center (DZL), Giessen, Germany

**Keywords:** very low birth weight infants, influenza, human, sepsis, outcome, periventricular leukomalacia, seasonality

## Abstract

**Objective:**

We investigated the relationship between influenza seasonality and outcome of very low birth weight infants (VLBWI) in a large observational cohort study of the German Neonatal Network.

**Materials and methods:**

Within the observational period (July 2009 until December 2014), five influenza seasons occurred (mean duration: 97 days, range: 48–131 days). We stratified VLBWI (*n* = 10,187) according to date of birth into three categories: (1) before influenza season, (2) during influenza season, and (3) 3 months after the end of the respective season. Outcomes were assessed in univariate and logistic regression analyses. In a subgroup of infants (*n* = 1497), the number of respiratory infections during the first 24 months of life was assessed.

**Results:**

VLBWI born during influenza season carried a higher risk for clinical sepsis (31.0 vs. 28.2%; *p* = 0.014) and periventricular leukomalacia (PVL, 3.7 vs. 2.5%, *p* = 0.004). In a multivariate logistic regression model, birth during influenza season was associated with PVL [odds ratio (OR) 1.47 (1.11–1.95), *p* = 0.007] and clinical sepsis [OR 1.13 (1.01–1.27), *p* = 0.036], independent of known risk factors, i.e., gestational age, multiple birth, gender, and small for gestational age. The risk for bronchopulmonary dysplasia was not influenced by influenza seasonality. In the small subgroup with information on 24 months follow-up (*n* = 1497), an increased incidence of common cold and bronchitis episodes was noted in infants born during influenza season.

**Conclusion:**

Our observational data indicate that preterm birth during influenza season is associated with PVL and sepsis. These are novel aspects that deserve further investigations to address underlying causes and to include virus surveillance.

## Introduction

Influenza infection can severely affect maternal health and pregnancy outcome (1). Outbreak reports on newly emerging influenza strains, e.g., 2009 H1N1 influenza A pandemic, clearly indicated that pregnant women are at an increased risk for hospitalization, acute respiratory distress syndrome, and death as compared to the general population (2). Influenza infection is known to trigger preterm birth, but the potential mechanism of influenza-mediated immune activation causing labor has not yet been clearly defined (3). Direct vertical transmission of the influenza virus to the fetus is rare but has been postulated to cause fetal demise or early neonatal death (4, 5). Hence, pregnant women constitute are target group for which specific preventive measures are required (6). Seasonal influenza epidemics are a driver of seasonality in gestation length (7). Seasonal influenza vaccines are effective interventions, as immunized mothers have a reduced risk to deliver prematurely during influenza season than unvaccinated mothers. In addition, maternal influenza immunization reduces the burden of febrile respiratory illness in young infants (8, 9). In Germany, seasonal influenza vaccination is recommended for all pregnant women from the second trimester and for pregnant women with underlying chronic disease from the first trimester since August 2010. Despite national recommendations for influenza vaccination of pregnant women, however, vaccination rates in European countries are low (≈10–26%) (10, 11). Influenza therefore remains a significant health-care problem during pregnancy with estimated incidence density rates of 1.8/100 person-weeks (11). It is yet unknown whether influenza seasonality affects the outcome of extremely preterm infants.

In the German Neonatal Network (GNN), we performed a large observational study including five influenza seasons to evaluate outcome data of very low birth weight infants (VLBWI) stratified to date of birth, i.e., before season, during season, or 3 months after the end of season. Two hypotheses prompted our study: infants born during season have an increased risk for clinical sepsis during primary stay in hospital and for respiratory infections during infancy as compared to infants born before season.

## Materials and Methods

The GNN is a population-based cohort study of VLBWI enrolled in 54 neonatal intensive care units in Germany (GNN). The data for this observational investigation were collected between the July 13, 2009 (3 months after influenza season 2008/2009 had finished) until the December 31, 2014. The inclusion criteria were as follows: birth weight <1500 g and gestational age ≥22 + 0 and <37 + 0 weeks. Infants with lethal abnormalities were excluded. After written informed consent by the parents, the attending physicians enrolled the infants in the GNN. Then, a predefined clinical data set of 220 parameters were recorded on case report forms and sent to the GNN coordinating center in Lübeck (see Parameters on Clinical Record Files for Infants Enrolled in the German Neonatal Network in Appendix). For those infants born in participating GNN centers but not enrolled in GNN, a basic data set including birth weight, gestational age, and major outcomes such as sepsis, bronchopulmonary dysplasia (BPD), and death was also collected. Reasons for non-participation in GNN included early death of infant, language difficulties, not approached for participation, and declined consent.

A physician specialized in neonatology or a study nurse monitored the data quality by annual site visits.

### Study Hypothesis

We hypothesized that infants born during influenza season have a higher likelihood to suffer from clinical sepsis (primary endpoint) than infants born before season. We additionally aimed to investigate whether influenza seasonality is associated with inflammation-mediated disorders, specifically periventricular leukomalacia (PVL) and BPD as secondary endpoints.

### Definitions

#### Influenza Season

The duration of an influenza season was based on data of the German sentinel surveillance system for influenza (Arbeitsgemeinschaft Influenza) at the Robert Koch Institute. For each calendar week, the positivity rate was calculated, i.e., the proportion of sentinel samples that were tested positive for influenza virus by PCR in the National Reference Center for Influenza. The start of an influenza season was defined as the first of two consecutive weeks, in which the lower 95% confidence limit of the positivity rate was at least 10%. The end of the epidemic period was determined by the week that precedes the first two consecutive weeks, in which the lower 95% confidence limit of the positivity rate drops below 10% [Table 1; (12, 13)]. Accordingly, “before influenza season” was defined as period from the first day after 3 months after the end of last season until the last day before the new season started. We also included a post-seasonal period of 3 months, defined as first day after the end of season until three full months after the end of season.

**Table 1:** **Influenza season**.

Influenza season	Before season	Season	3 months after season
October 12, 2009–January 31, 2010 (days)	90^a^	111	90
December 13, 2010–April 10, 2011 (days)	226	118	92
February 6, 2012–April 22, 2012 (days)	211	76	92
December 10, 2012–April 21, 2013 (days)	140	131	92
February 17, 2014–April 6, 2014 (days)	210 + 178^b^	48	92
Total number of days	1055	484	458
Number of VLBWI	7552	3148	3537

*^a^Influenza season 2008/2009: December 1, 2008 until April 12, 2009*.

*^b^July 6, 2014–December 31, 2014, influenza season 2014/2015: January 5, 2015–April 19, 2015*.

*Influenza season was defined according to data of the German sentinel surveillance system for influenza (Arbeitsgemeinschaft Influenza) at the Robert Koch Institute (12, 13)*.

### Clinical Characteristics and Outcomes

The primary outcome *Clinical sepsis* was defined as sepsis with at least two signs (temperature >38°C or <36.5°C, tachycardia >200/min, new onset or increased frequency of bradycardias or apneas, hyperglycemia >140 mg/dl, base excess <−10 mval/l, changed skin color, increased oxygen requirements) and one laboratory sign (C-reactive protein >1 mg/dl, immature/neutrophil ratio >0.2, white blood cell count <5/nl, platelet count <100/nl) and antibiotic treatment for ≥5days, but no proof of causative agent in the blood culture.

*Gestational age* was calculated from the best obstetric estimate based on early prenatal ultrasound and obstetric examination. Small for gestational age was defined as birth percentile <10 according to gestational age.

*Blood culture confirmed sepsis* was defined as clinical sepsis with proof of causative agent in the blood culture.

*Bronchopulmonary dysplasia* was diagnosed when needing supplemental oxygen at 36 weeks of post-menstrual age (BPD).

*Intracerebral hemorrhage (ICH)* grades I–IV were diagnosed according to the ultrasound criteria of Papile (14) in line with a standardized protocol derived from the DEGUM (German Society for Ultrasound in Medicine).

*Periventricular leukomalacia* was defined as white matter brain injury, characterized by cystic degeneration of white matter near the lateral ventricles as diagnosed by ultrasound imaging which was applied in all participating centers.

*Severe complication* was defined as diagnosis of at least one of the following outcome measures: ICH grade III or intracerebral parenchymal hemorrhage, PVL, retinopathy of prematurity (ROP) requiring surgery, necrotizing enterocolitis, or focal intestinal perforation requiring surgery or need for ventriculoperitoneal shunting.

*Death* was defined as death occurring after admission to NICU within the primary stay in hospital.

### Follow-up

German Neonatal Network infants are followed up regularly by the GNN study team at the age of 5 years with physical examinations and neurodevelopmental testings on-site. Neurodevelopmental testing included Movement Assessment Battery for Children-2 (M-ABC-2), Wechsler Preschool and Primary Scale of Intelligence-III (German version), visual testing and audiometry.

For the 24-month follow-up, parents of surviving infants enrolled in GNN (birth year 2009–2011, *n* = 3946) received a voluntary questionnaire (according to KiGGS survey 1–2 years from Robert Koch Institute, Germany) (15). The KiGGS survey (German Health Interview and Examination Survey for Children and Adolescents) was conducted to collect representative data on the health status of children in Germany.

### Statistical Analysis

In order to avoid selection bias, we analyzed the whole cohort of infants born in GNN centers and the subgroup of infants enrolled in GNN. We stratified VLBWI according to date of birth into three categories: (1) born before influenza season, (2) born during influenza season, and (3) born in the 3 months directly following the end of the season.

We modeled the probability 1 vs. 0 of suffering from a condition, i.e., clinical sepsis, in relation to influenza seasonality, i.e., exposure time which is defined by the annual reports on influenza surveillance data in Germany (Robert Koch Institute, Berlin). Data analysis was performed using the SPSS 22.0 data analysis package (Munich, Germany). Differences between infants born before, during, and 3 months after season were evaluated with Pearson chi-square test, *T* test, and Mann–Whitney *U* test. To determine potential associations between influenza seasonality and outcome of VLBWI, we conducted multiple logistic regression analyses with known confounding variables for outcomes, i.e., gestational age, gender, multiple birth, SGA, and exposure to antenatal steroids. Odds ratios (OR) and 95% confidence intervals (CI) were calculated. A *p* value of <0.05 was considered statistically significant. Missing data were not imputed.

### Ethics

The study was approved by the ethics committee of the University of Lübeck (08-022) and the local ethical committees at each study center. Written informed consent was obtained from at least one parent on behalf of the infant enrolled in the GNN.

## Results

### Influenza Seasons

Within the observational period (July 13 until December 31, 2014), five influenza seasons occurred (mean duration: 97 days, range: 48–131 days). About 10,187 VLBWI were enrolled in GNN, *n* = 5449 before influenza season, *n* = 2224 during influenza season, and *n* = 2514 up to 3 months after the end of a season (Table 1). The maternal background in GNN enrolled infants was German (74.6%), other Europe/Russia (10.5%), Middle East/Turkey (7.5%), and Asia or Africa (6.4%).

### Short-term Outcomes

#### Univariate Analysis

We found no remarkable differences in clinical characteristics within categories. A higher risk for clinical sepsis was noted in infants born during influenza season as compared to infants born before season (31.0 vs. 28.2%; *p* = 0.014; Table 2). Preterm birth during influenza season was also associated with a higher risk for PVL (3.7 vs. 2.5%, *p* = 0.004) as compared to infants born before season, while the risk for BPD was unaffected by influenza seasonality. We alternatively assessed date of birth according to month or quarterly period of the year (January–March, April–June, July–September, and October–December) and link to PVL. We found no association between PVL and month of birth (mean 3.1%, range 2.6–4.2%, *p* = 0.19) or quarterly period of the year (mean 3.1%, range 2.8–3.7%, *p* = 0.1).

**Table 2:** **Clinical characteristics of VLBWI according to timely relation with influenza season**.

Clinical characteristics	All	Before season	Influenza season	3 months after season	p^1^	p^2^
Number of infants	10,187	5449	2224	2514		
Gestational age (weeks), mean (SD)	28.7 (2.7)	28.7 (2.7)	28.7 (2.8)	28.7 (2.7)	0.9^#^	0.7*
Birth weight (g), mean (SD)	1059 (307)	1061 (307)	1055 (307)	1057 (307)	0.1^#^	0.3*
SGA (%)	18.5	18.4	20.1	17.3	0.05	0.08
Gender, female (%)	48.8	49.6	47.7	48.1	0.2	0.1
Multiple birth (%)	34.6	35.5	33	34.1	0.1	0.04
Clinical sepsis (%)	29.2	28.2	31	29.5	0.05	0.014
Blood culture proven sepsis (%)	12.1	12.1	12.5	12	0.9	0.6
BPD (O_2_ at 36 weeks, %)	12.8	12.3	13.4	13.4	0.2	0.2
Supplemental O_2_ at discharge	3.7	3.7	4	3.5	0.7	0.6
Intracerebral hemorrhage (%)	16.6	16.5	17.1	16.2	0.7	0.5
PVL (%)	3.0	2.5	3.7	3.4	0.009	0.004
Severe complication (%)	22.3	21.8	23.3	22.4	0.4	0.2
Death (%)	3.6	3.4	4.0	3.5	0.4	0.2
BPD (O_2_ at 36 weeks) or death (%)	15.1	14.6	16.0	15.5	0.2	0.1

p Values are derived from Pearson chi^2^ test or t-test (^#^) or Mann–Whitney U-test if indicated (*), p^1^ comparison of all three categories, p^2^ infants born during season vs. infants born before season.

#### Logistic Regression Analysis

We further assessed clinical sepsis and PVL in relation to influenza seasonality in a multivariate logistic regression model including known confounding variables, i.e., gestational age, gender, multiple birth, SGA, and exposure to antenatal steroids. Birth during influenza season was associated with PVL [OR 1.47 (1.11–1.95), *p* = 0.007] and clinical sepsis [OR 1.13 (1.01–1.27), *p* = 0.036]. Infants born 3 months after the end of season also had a higher predisposition to PVL, but not for clinical sepsis as compared to infants born before influenza season (Table 3). To account for cardiovascular compromise as potential risk factor for PVL, we included the need of inotropes in the first 24 h in our model. This proved to be significantly associated with PVL [OR 1.84 (1.34–2.52), *p* < 0.001] while birth during influenza season also remained a stable independent risk factor [OR 1.51 (1.08–2.11), *p* = 0.017]. Likewise, clinical sepsis was associated with development of PVL [OR 1.74 (1.35–2.24), *p* < 0.001] which did not affect the impact of birth during influenza season [OR 1.45 (1.1–1.93), *p* = 0.01]. The link between influenza seasonality and PVL risk was not influenced by causes of preterm birth, i.e., amniotic infection [OR 1.1 (0.84–1.44), *p* = 0.5]; birth during influenza season [OR 1.47 (1.11–1.95), *p* = 0.008] or prolonged premature rupture of membranes [PPROM; OR 0.93 (0.72–1.21), *p* = 0.6]; birth during influenza season [OR 1.48 (1.11–1.97), *p* = 0.007].

**Table 3:** **Multivariate logistic regression analysis (infants enrolled in GNN)**.

Outcome	Clinical sepsis	PVL
Affected infants/controls	2890/7044	297/9616
Gestational age/week	OR 0.73 (0.71–0.74), *p* < 0.001	OR 0.79 (0.75–0.82), *p* < 0.001
Gender, female	OR 0.9 (0.82–0.98), *p* < 0.025	OR 0.91 (0.72–1.15), *p* < 0.4
Multiple birth	OR 0.89 (0.8–0.98), *p* < 0.022	OR 0.97 (0.76–1.25), *p* < 0.8
Antenatal steroids	OR 0.8 (0.69–0.94), *p* < 0.005	OR 0.57 (0.41–0.78), *p* < 0.001
Small for gestational age	OR 1.82 (1.6–2.06), *p* < 0.001	OR 0.9 (0.64–1.25), *p* < 0.5
Born influenza season	OR 1.13 (1.01–1.27), *p* < 0.036	OR 1.47 (1.11–1.95), *p* < 0.007
Born 3 months after	OR 1.05 (0.93–1.17), *p* < 0.4	OR 1.33 (1.0–1.76), *p* < 0.046

*Data are given as odds ratios OR (95% confidence interval). Date of birth in relation to influenza seasonality was included as categorical variable*.

*ORs were calculated including date of birth before season as reference*.

### Follow-up

**Figure 1:**
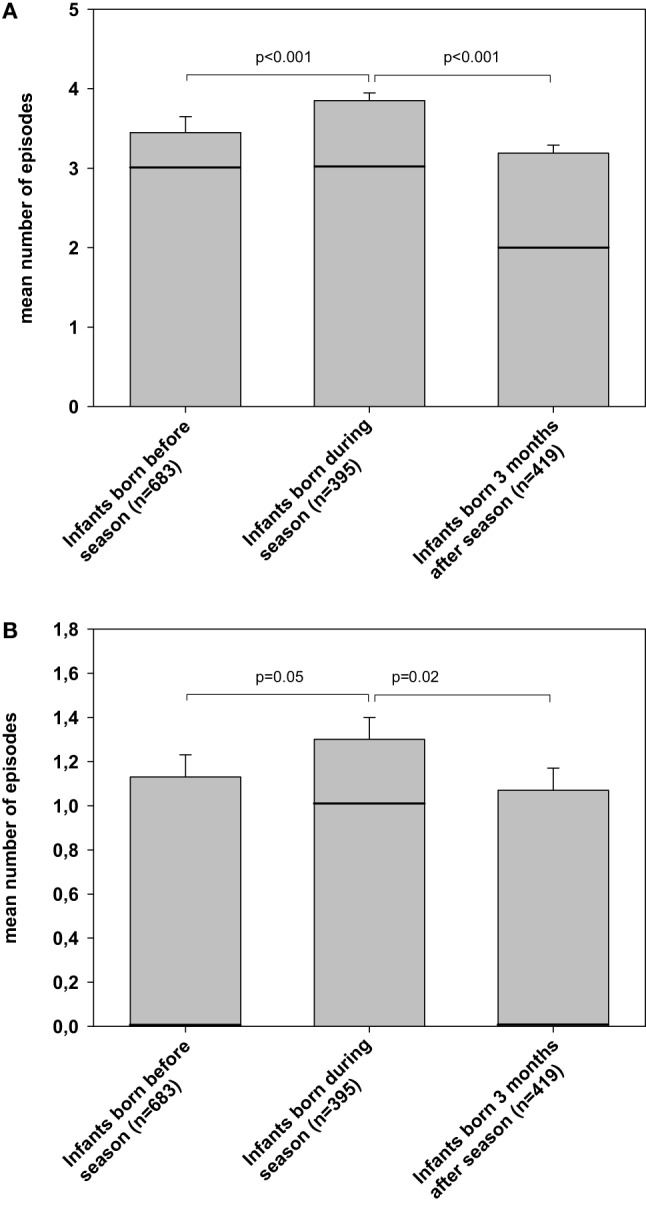
**Risk for bronchitis and common cold in the first 24 months after discharge**. The figure describes the mean numbers of episodes (bars, SEM as error bars, solid line as median) for **(A)** common cold and **(B)** bronchitis occurred during infancy. These data are based on parents’ responses to the KIGGS questionnaire (German Health Interview and Examination Survey for Children and Adolescents; www.kinder-jugend-gesundheit21.de, page 8, question 23) at 24 months of age. Data are described according to date of birth, i.e., before influenza season, during influenza season, and 3 months after the respective season had finished, respectively.

In a small subgroup, if surviving infants born 2009–2011 (*n* = 1497/3946, response rate by questionnaire: 37.9%) data on 24-month follow-up were available. In this subgroup, 84.5% of responding parents had a German background, while 8% mothers were born in other European countries and Russia, 4.5% in the Middle East/Turkey, and 2.6% Asia or Africa. VLBWI born during influenza season (*n* = 395) were more often affected from upper airway infection (65 vs. 57 vs. 48%, *p* = 0.001) than infants born before (*n* = 683) or 3 months after the end of season (*n* = 419), respectively. VLBWI born during influenza season also had a higher number of episodes of common cold as compared to infants born before season or 3 months thereafter (mean/median ± SE; 3.9/3 ± 0.1 vs. 3.5/3 ± 0.2 vs. 3.2/2 ± 0.1 episodes, *p* = 0.006; Figure 1A) and for bronchitis (1.3/1 ± 0.1 vs. 1.1/0 ± 0.1 vs. 1.1/0 ± 0.1 episodes, *p* = 0.04; Figure 1B). No significant differences were noted for atopic dermatitis between VLBWI born during season as compared to birth before or 3 months after season (8.5 vs. 6.9 vs. 6.3%, *p* = 0.3).

## Discussion

To the best of our knowledge, this is the first large scale population-based data on the link between influenza seasonality and outcome of VLBWI. Preterm birth during influenza season was associated with a higher risk of PVL and clinical sepsis.

Influenza infection of the pregnant mother is a well-known risk factor for preterm delivery which proved to be independent of socio-economic factors (7). However, the outcome of VLBWI born during influenza season has received little attention so far, as most reports on outcome after antenatal influenza exposure restrict their data to short-term perinatal outcome, i.e., birth weight, gestational age, Apgar scores, and neonatal death (6, 16). We hypothesized that influenza seasonality affects the outcome of VLBWI. To address this aspect, we modeled the probability of a certain outcome and compared infants born and exposed during influenza season to those born either before or 3 months after the end of influenza season. The latter category was included to account for the fact that maternal antibodies (after exposure during season or previous vaccination) may have transferred *in utero* to those infants born up 3 months after the end of a season. Our approach is limited by the use of “potential influenza exposure (season)” as surrogate measure rather than definite virological surveillance and vaccination status in mothers of VLBWI and their offspring. Such surveillance has not yet been introduced into clinical routine in NICUs or experimental studies in large cohorts.

Very low birth weight infants frequently suffer from clinical sepsis, i.e., an incidence of 25–30% in the GNN cohort. The risk profile is characterized by gestational age, immaturity of systemic, and local immune responses and necessity of invasive procedures. The symptoms of clinical sepsis are non-specific, often indistinguishable between viral and bacterial illness. Due to the diagnostic challenge, antibiotic therapy is often started for suspected sepsis, but the cause of clinical deterioration often remains uncertain (17). In our study cohort, VLBWI born during influenza season had an increased risk for clinical sepsis. In temperate climates, influenza is thought to exist at a low level of intensity throughout the year but exhibits a marked seasonal increase, typically during the winter months (18). Our observation may just reflect epidemiological characteristics of viral illness including influenza which drive exposure for mothers and their infants, as well as health-care workers (19). Other environmental factors may add to clinical sepsis risk during influenza season, particularly understaffing, respiratory illness of medical staff and overcrowding, which cannot be evaluated in our data set. Given the specific susceptibility of pregnant women during influenza season, the complex interplay of the mother’s immune response to influenza and fetal sensing of local microbial milieu might also increase the infection risk in the offspring. To address this aspect, we evaluated causes of preterm delivery but found no differences in relation to influenza seasonality. Infants born during season also had a higher predisposition to common cold and bronchitis in the first 24 months of life as compared to those infants born before or 3 months after season. This observation might be related to long-lasting reduced local immunity in infants born during influenza season. On the other hand, protective maternal antibody transfer to those infants born after season may play a role. It should be noted that the subgroup of infants with parental information on infections during infancy is small, 37.9% response rate to voluntary questionnaires. Following aspects may have contributed to the low response rate. First, the questionnaire contains many questions, hence motivation and time of parents to fill out the forms might be limited. Second, tracking parents is difficult for changing addresses. We contacted the German registration offices and send our questionnaires to the new addresses but still had a poor feedback. Third, language problems might account for our observation, as responding families had more often German background as compared to the whole cohort (84.5 vs. 74.6%). Therefore, the validity of parents’ feedback is limited in our setting. However, our data are hypothesis generating and a useful starting point for an understanding of illness attributable to respiratory infection (20).

Our data imply an increased risk for classical cystic PVL in VLBWI infants born during influenza season. This is a novel aspect that deserves further investigation with specific attention to the multi-factorial pathogenesis of PVL. Patients with PVL often develop cerebral palsy, seizures, and vision or hearing deficits (21). There is general consensus that development of PVL may be caused by (1) decreased blood flow (ischemia) or oxygen supply (hypoxia) of the periventricular white matter region and (2) damage to glial cells due to inflammation. These pathogenic conditions may occur ante-, peri-, and postnatally with varying underlying causes, including placenta dysfunction, fetal distress, and infections. As a result, a sequence of inflammatory responses is set off, including the release of cytokines and free radicals which are toxic to the developing brain (22). Influenza infection may cause neurological sequelae that are clinical correlates of hypoxic–ischemic and inflammatory signaling cascades. For example, the Vascular Effects of Infection in Pediatric Stroke study demonstrated that upper airway infection transiently increased the risk of childhood ischemic stroke (23). In addition, acute encephalopathies with imaging signs of PVL were reported after influenza A infection in children during H1N1 2009 pandemic, as well as cases of (meningo)-encephalitis (24). Presumably, these adverse outcomes are immune mediated rather than a consequence of influenza virus directly entering the central nervous system (CNS). In a Japanese surveillance study over nine influenza seasons, only 10% of patients with neurological complications had PCR detection of influenza virus in the cerebrospinal fluid (25). To account for potential patho-mechanisms of PVL in our cohort, we included surrogates for cardiovascular compromise (need for inotropes) or inflammation (clinical sepsis, preterm labor, amniotic infection, and PPROM) in regression models. Influenza seasonality, however, remained a stable independent risk factor of PVL. The same holds true when basic data sets of all VLBWI born in GNN centers (total *n* = 14,237 VLBWI, *n* = 7552 born before influenza season, *n* = 3148 born during influenza season, *n* = 3537 born up to 3 months after the end of a season), including those who were not enrolled in GNN, were evaluated. In all VLBWI, preterm birth during influenza season was associated with a higher risk for PVL (3.8 vs. 2.7%, *p* = 0.005) as compared to infants born before season. Logistic regression analysis also revealed an association between birth during influenza season and PVL [OR 1.39 (1.1–1.75), *p* = 0.007]. Immunization status of the mother was unknown in our study. Theoretically, an effect of vaccination against influenza during pregnancy might be assumed. Further studies should take this point into regard.

It is current clinical standard to diagnose PVL in sequential head ultrasound exams in preterm infants but sensitivity is limited. Future studies need to include MRI technology in order to detect subtle white matter changes (punctuate lesions) and diffuse white matter injury (diffuse excessive high signal intensity, DEHSI) (26).

Our observational data point to the role of prevention. As of yet, the benefit of influenza vaccinations of pregnant mothers and infants is not clear in the population of VLBWI. Particularly, the effectiveness of influenza vaccination in preterm infants remains uncertain (27). Previous data noted an adequate immunogenicity in 6- to 17-month-old extremely low birth weight infants after two influenza vaccine doses (28). Further vaccination studies along with experimental models are needed to address causal relationships between seasonal influenza and adverse outcome (29, 30). In order to protect highly vulnerable infants, influenza vaccination of medical staff should be enforced.

The major strengths of our study include a large population-based cohort of VLBWI which is well phenotyped with quality assurance of data by on-site monitoring. Our study has limitations. The stratification in date of birth categories in relation to influenza seasonality is based on epidemiological data from a national reference center rather proven evidence of influenza in VLBWI and their mothers. We defined birthday in relation to influenza seasonality as the main parameter for definition of the population at risk. Alternatively, we assessed date of birth according to month and found no association to PVL. Our observation is not a causal relationship and other respiratory viruses with seasonal patterns may have contributed to our finding. Virological surveillance in future population-based studies is needed and might provide data relevant to the development of age-specific prevention strategies.

In conclusion, our observational data indicate that preterm birth during influenza season is associated with adverse outcome. This is a novel aspect that deserves further investigations to address underlying causes and to include virus surveillance.

## Author Contributions

CH wrote the first draft of the manuscript, and no honorarium, grant, or other forms of payment were given to anyone to produce the manuscript. Each author listed on the manuscript has seen and approved the submission of this version of the manuscript and takes full responsibility for the manuscript. CH and WG conceptualized and designed the study, supervised and coordinated the data collection, carried out the data analysis, drafted the initial manuscript, and approved the final manuscript as submitted. AH, JR, MK, and EH conceptualized and designed the study, supported data collection, drafted the initial manuscript, and approved the final manuscript as submitted. CH, DV, AS, and TO coordinated and supervised data collection at their sites, supported the study design and the development of data collection instruments, critically reviewed the manuscript, and approved the final manuscript as submitted.

## Conflict of Interest Statement

The authors declare that the research was conducted in the absence of any commercial or financial relationships that could be construed as a potential conflict of interest.

## Appendix

### A. Parameters on Clinical Record Files for Infants Enrolled in the German Neonatal Network

#### Birth Data of the Enrolled Infant

Initials, date of birth, inborn/outborn, gender, multiple birth, feto-fetal transfusion syndrome and therapy, causes of premature birth (premature labor, chorioamnionitis, gestosis, HELLP syndrome, conspicuous CTG, abruption of placenta, anhydramnion, premature rupture of membranes >5 days/timepoint, onset before/with contractions, and anhydramnios), mode of birth (spontaneous, elective section, and emergency section), APGAR 5′, APGAR 10′, pH of umbilical artery, base excess of umbilical artery, birth weight, length, circumference of head, body temperature at birth, gestational age in weeks + days, and congenital anomalies (life threatening, letal, and CRIBS score).

#### Resuscitation and Support within First 60 min of Life

Timepoint of resuscitation, sustained inflation, less invasive surfactant application (LISA), surfactant *via* endotracheal tube, intubation, bicarbonate infusion, application of volume, adrenalin, cardiopulmonary resuscitation, lactate levels within first 60 min, lowest mean arterial pressure within first 24 h, and use of inotropes within first 24 h.

#### Maternal Data

Age, ethnicity, gravida, para, antenatal corticosteroids (type, completed cycle), tocolysis, cerclage, pessar, progesterone, admission to hospital >1 week before birth, and antenatal antibiotics (agents listed).

#### Individual Therapy of the Infant

Prophylactic drugs (vitamin K, teicoplanin/vancomycin, fluconazole, indomethacin, erythropoietin, and others), antibiotic therapy with specification of given antibiotics (agents listed), specification of inotrope therapy (dopamine, noradrenaline, dobutamine, adrenaline), analgetics (acetaminophen, morphine, thiopental, pentobarbital, midazolam, fentanyl, phenobarbital, chloral hydrate, piritramide, sufentanil, propofol, diazepam, and others), surfactant (including number of applications), mode of surfactant application, diuretics (furosemide, hydrochlorothiazide, and spironolactone), inhalative medicaments (salbutamol, NO, budesonide, ipratropium bromide, fluticasone, and others), transfusions (amount of red blood cell, platelet and fresh frozen plasma transfusions), caffeine, theophylline, doxapram, and other drugs, such as acetylcysteine, ambroxol, calcium gluconate, calcium glycerophosphate, calcium phosphate, dexamethasone, iron, glucose, hydrocortisone, ibuprofen, indomethacin, insulin, l-thyroxin, natrium gluconate, natrium glycerophosphate, bicarbonate, NaCl, omeprazole, phosphate, prednisolone, ranitidine, sildenafil, ursodeoxycholic acid, vitamins, zinc, and others, probiotics (*Lactobacillus* + *Bifidobacte rium, Lactobacillus* GG, *E. coli*, and others), vaccination (hexa polyvalent vaccines, pneumococcal vaccines, palivizumab, and rotavirus vaccination), stopping of application of medicaments due to adverse events, used central venous lines (central venous catheter, umbilical cord catheter, including material of catheter, silicone, and polyurethane) and possible complications, arterial catheter (umbilical artery catheter, peripheral artery catheter), complications of central access (infection, thrombosis), start of oral feeding, time to reach 150 ml/kg oral feeding in days, length of stay of intravenous catheter, and type of oral nutrition during hospital stay and at discharge (breast milk, donated breast milk, special preterm milk, industrial milk, and fortification of milk), begin and ending of oxygen supply, oxygen supply at discharge, and CPAP (CPAP, CPAP with IMV, HFO-CPAP, highflow nasal cannula), type of ventilation [conventional ventilation (SIMV, IMV, etc.), high frequency ventilation], time intervals of invasive ventilation, date of completed ventilation supply (inclusive CPAP), pulmonary interstitial emphysema, pneumothorax (during invasive ventilation, non-invasive ventilation, spontaneous breathing), drainage of pneumothorax, sepsis (pathogen) within/after 72 h of admission, pneumonia within/after 72 h of admission, necrotizing enterocolitis II/III, cranial ultrasound (grade of intracerebral hemorrhage I–IV, periventricular leukomalacia), hip dysplasia (grade Graf), retinopathy of prematurity (grades 0–5) therapy of retinopathy of prematurity (bevacizumab, laser, and cryotherapy), result of screening for hearing loss, date and type of surgery (PDA, NEC, FIP, VP-shunt, herniotomy, and others), bronchopulmonary dysplasia (Walsh), date of discharge, weight, length, and head circumference at discharge, discharge home or to another facility, cause of death.
