# Supplementary material for: Preterm Birth during Influenza Season Is Associated with Adverse Outcome in Very Low Birth Weight Infants
Source: Front Pediatr. 2016 Nov 30;4:130. doi: 10.3389/fped.2016.00130 (PMC5129678; doi:10.3389/fped.2016.00130)
Supplement: Supplementary file 1 [file Table_1.DOCX]

**Suppl table 1 Clinical characteristics of VLBWI according to timely relation with influenza season (all infants born in GNN centers)**

| **Clinical characteristics** | **all** | **Before season** | **Influenza season** | **3 mo. after** | **p^1^** | **p^2^** |
| --- | --- | --- | --- | --- | --- | --- |
| Number of infants | 14237 | 7552 | 3148 | 3537 |  |  |
| Gestational age (weeks), mean (SD) | 28.7 (2.9) | 28.7 (2.9) | 28.7 (2.9) | 28.7 (2.9) | 0.7# | 0.9* |
| Birth weight (g), mean (SD) | 1056 (316) | 1058 (317) | 1053 (316) | 1056 (315) | 0.4# | 0.3* |
| SGA (%) | 20.2 | 20.3 | 21.3 | 19.0 | 0.07 | 0.3 |
| Gender, female (%) | 48.5 | 48.8 | 48.1 | 48.2 | 0.8 | 0.5 |
| Multiple birth (%) | 32.9 | 33.8 | 31.7 | 32.1 | 0.05 | 0.03 |
| Clinical sepsis (%) | 29.1 | 28.5 | 29.7 | 30.0 | 0.2 | 0.2 |
| Blood-culture proven sepsis (%) | 10.9 | 10.8 | 11.0 | 11.2 | 0.8 | 0.8 |
| BPD (O_2_@36 weeks, %) | 12.3 | 11.9 | 12.6 | 12.7 | 0.5 | 0.4 |
| Intracerebral hemorrhage (%)  PVL (%) | 17.5  3.1 | 17.4  2.7 | 17.7  3.8 | 17.7  3.4 | 0.9  0.01 | 0.7  0.005 |
| Severe complication (%) | 23.5 | 22.9 | 24.2 | 24.4 | 0.2 | 0.2 |
| Death (%)  BPD (O_2_@36 weeks) or death (%) | 9.4  19.7 | 9.4  19.5 | 9.6  20.2 | 9.1  19.8 | 0.8  0.7 | 0.8  0.4 |
| Enrolled in GNN (%) | 71.6 | 72.2 | 70.6 | 71.1 | 0.8 | 0.9 |

p-values are derived from Pearson-chi^2^ test or T-test (#) or Mann-Whitney-U-test if indicated (*), p^1^ comparison of all three categories, p^2^ infants born during season vs. infants born before season
