# Supplementary material for: Preterm Birth during Influenza Season Is Associated with Adverse Outcome in Very Low Birth Weight Infants
Source: Front Pediatr. 2016 Nov 30;4:130. doi: 10.3389/fped.2016.00130 (PMC5129678; doi:10.3389/fped.2016.00130)
Supplement: Supplementary file 2 [file Table_2.DOCX]

**Suppl Table 2 Multivariate logistic regression analysis (all infants born in GNN centers)**

| **Outcome** | **Clinical sepsis** | **PVL** |
| --- | --- | --- |
| Number of affected infants/controls | 3232 / 7892 | 429 / 13335 |
| Gestational age per week | OR 0.74 (0.73-0.75), p<0.001 | OR 0.8 (0.77-0.83), p<0.001 |
| Gender, female | OR 0.9 (0.82-0.98), p=0.02 | OR 0.88 (0.72-1.07), p=0.2 |
| Multiple birth | OR 0.87 (0.8-0.96), p=0.005 | OR 0.92 (0.75-1.15), p=0.5 |
| Small-for-gestational age | OR 1.7 (1.51-1.91), p<0.001 | OR 0.97 (0.75-1.26), p=0.8 |
| Born during influenza season  Born 3 months after influenza season | OR 1.06 (0.95-1.18), p=0.3  OR 1.06 (0.96-1.18), p=0.3 | OR 1.39 (1.1-1.75), p=0.007  OR 1.25 (0.99-1.57), p=0.07 |
